# Levels of Physical Activity at Age 10 Years and Brain Morphology Changes From Ages 10 to 14 Years

**DOI:** 10.1001/jamanetworkopen.2023.33157

**Published:** 2023-10-05

**Authors:** Fernando Estévez-López, Lorenza Dall’Aglio, María Rodriguez-Ayllon, Bing Xu, Yueyue You, Charles H. Hillman, Ryan L. Muetzel, Henning Tiemeier

**Affiliations:** 1 Department of Social and Behavioral Sciences, Harvard T. H. Chan School of Public Health, Boston, Massachusetts; 2 Department of Education, Faculty of Education Sciences, SPORT Research Group and CERNEP Research Center, University of Almería, Almería, Spain; 3 Department of Child and Adolescent Psychiatry/Psychology, Erasmus MC University Medical Centre, Rotterdam, the Netherlands; 4 The Generation R Study Group, Erasmus MC University Medical Centre, Rotterdam, the Netherlands; 5 Department of Epidemiology, Erasmus MC University Medical Centre, Rotterdam, the Netherlands; 6 Department of Public Health, Erasmus MC University Medical Centre, Rotterdam, the Netherlands; 7 Department of Psychology, Northeastern University, Boston, Massachusetts; 8 Department of Physical Therapy, Movement and Rehabilitation Sciences, Northeastern University, Boston, Massachusetts; 9 Department of Radiology and Nuclear Medicine, Erasmus MC University Medical Centre, Rotterdam, the Netherlands

## Abstract

**Importance:**

Physical activity may promote healthy brain development in children, but previous research was predominantly cross-sectional and included small samples, providing limited knowledge.

**Objective:**

To investigate the longitudinal associations of physical activity with brain morphology changes.

**Design, Setting, and Participants:**

A 4-year longitudinal population-based cohort study in Rotterdam, the Netherlands, embedded in Generation R, a cohort from fetal life onward. From the women enrolled during pregnancy, children who had repeated measures of brain structure at ages 10 (range 8 to 12) years and 14 (range 13 to 15) years were included. Data were collected from March 2013 to November 2015 (baseline) and from October 2016 to January 2020 (follow-up). Data were analyzed from April to December 2022.

**Exposure:**

At age 10 years, both the child and their primary caregiver reported the child’s levels of physical activity with regard to sport participation, outdoor play, and total physical activity. Primary analyses were based on an average multi-informant report.

**Main outcomes and measures:**

Brain morphology was quantified by magnetic resonance imaging. Hypothesized regions of interest were the bilateral amygdala and hippocampal volumes. Global brain measures were studied to test the specificity of the hypothesis.

**Results:**

Data were available for 1088 children (566 girls [52%]; 693 [64%] Dutch). Their mean (SD) age at baseline was 10.1 (0.6) years. For amygdala volume change, positive associations with multi-informant reports of total physical activity (β = 2.6; 95% CI, 0.3-4.9) were found. Total physical activity was associated with hippocampal volume increases only when reported by the child (β = 3.1; 95% CI, 0.4-5.8). No robust associations with global brain measures were found.

**Conclusions and relevance:**

In this cohort study of 1088 children, more physical activity at 10 years was consistently associated with an increase in amygdala volume in children aged 10 to 14 years. Physical activity and increases in hippocampal volume were found using child reports of physical activity only. These findings suggest physical activity in late childhood was prospectively associated with volumetric changes in specific subcortical structures, but not to global brain development, from late childhood to early adolescence. These findings may inform the design of future public health interventions to best facilitate neurodevelopment with physical activity.

## Key Points

**Question:**

Is physical activity in late childhood associated with changes in brain morphology from late childhood to early adolescence?

**Findings:**

In this cohort study of 1088 children, participants who engaged in more physical activity at age 10 years showed larger increases in amygdala volume from ages 10 to 14 years; similar but less robust findings were observed in the hippocampus. No associations were found between physical activity and global brain morphology measures.

**Meaning:**

These findings suggest that during the transition from late childhood to early adolescence, physical activity may influence the neurodevelopment of subcortical areas that are plastic and may underlie cognition, emotion, learning, and psychiatric disorders.

## Teaser

This cohort study evaluates the association between physical activity in late childhood and changes in brain morphology from late childhood to early adolescence.

## Introduction

Physical activity is a major determinant of health.^1,2^ During the transition from late childhood to early adolescence, physical activity might play a role in neurodevelopment.^1^ Global brain volume, particularly gray matter, reaches its peak maturation by adolescence.^3^ However, many structures, such as the hippocampus and amygdala, continue to develop, remaining particularly sensitive to environmental exposures.^1,2,4^ Additionally, postnatal neurogenesis has been shown in these 2 regions.^5,6^ Similarly, total white matter volume continues to increase into adulthood.^7^ A better understanding of the longitudinal association of physical activity with brain structure may inform future public health interventions to promote healthy brain development with physical activity.

Research on the outcomes of physical activity and children’s brains remains scarce. Past research was predominantly cross-sectional,^8,9^ with only a few notable exceptions,^10,11,12^ and usually included subcortical structures, most often the hippocampus, as regions of interest.^2^ For instance, a previous cross-sectional investigation^8^ in 4191 children showed associations between higher sport participation and larger volume of the hippocampus. Importantly, this study did not include repeated measures of the brain, precluding the ability to test the temporality of the associations. The majority of prior longitudinal research on physical activity and the hippocampus focused on older adults.^2,13^ For example, a randomized clinical trial (RCT) of 120 participants demonstrated that physical activity reversed age–related volume loss in the hippocampus.^10^ In children, however, the current understanding regarding the longitudinal association of physical activity and volume of the hippocampus requires further investigation. Recently, an RCT^11^ found no effects of physical exercise on hippocampal volume in children aged 8 to 11 years with overweight or obesity. This negative finding may reflect the fairly low sample size (109 participants).^14^ The emerging field of population neuroimaging highlights the need to study large samples to achieve higher precision and generalizability.^14^ Longitudinal population-based investigations are required to obtain reproducible findings elucidating the temporal association between physical activity and neurodevelopment.

Similarly to the hippocampus, the amygdala is particularly relevant given its plasticity and susceptibility to environmental stressors during childhood.^4,15^ However, our understanding of such plasticity is limited in humans as the literature has traditionally focused on harmful exposures (eg, early life adversity).^4,15^ In animal models, physical activity induces neuroplasticity in the amygdala,^16^ which is in line with findings from investigations of the human hippocampus.^10,17^ Prior research on children’s physical activity has typically omitted the study of the amygdala, but given its functional roles and evidence from animal studies, it could constitute a key candidate for investigation. Collectively, understanding how to enhance the structural development of these 2 structures in children may contribute to the prevention of psychiatric problems later in life.^18,19,20^

This study comprehensively examined the longitudinal association of physical activity (reported by multiple informants and defined by several dimensions; ie, sport participation, outdoor play, and total physical activity) in late childhood with changes in brain morphology assessed from late childhood (age 10 years) to early adolescence (age 14 years) in the general population. According to prior literature, we hypothesized higher levels of physical activity in late childhood would be longitudinally associated with increases in the volume of the hippocampus^2,10,13,17^ and amygdala^4,15,16^ over a 4-year period.

## Methods

### Study Design and Participants

This research is embedded in the Generation R Study, a population-based cohort from fetal life onward. A detailed description of the cohort was published previously.^21^ Data collection was conducted between March 2013 and November 2015 (age 10 years visit) and between October 2016 and January 2020 (age 14 years visit).^22^ Magnetic resonance imaging (MRI) was acquired at both visits in a research-dedicated imaging facility. This study followed the Strengthening the Reporting of Observational Studies in Epidemiology (STROBE) reporting guideline.

**Figure 1.**
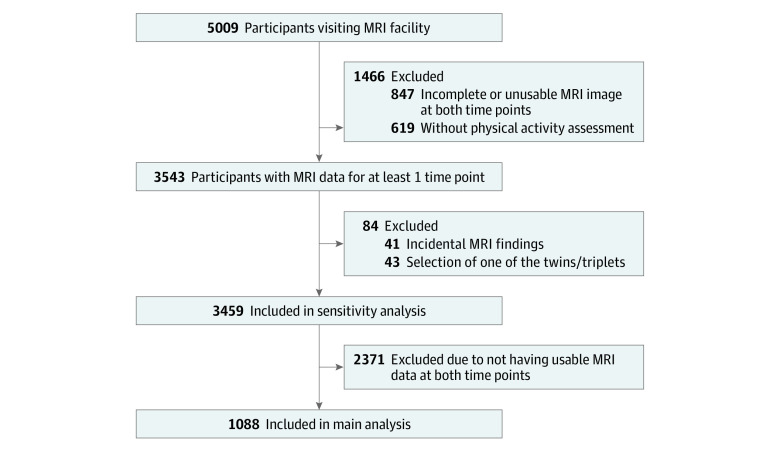
Flowchart of Participants MRI indicates magnetic resonance imaging.

Figure 1 provides the flowchart. The main sample of the study comprised participants who had repeated MRI data. For sensitivity analyses, we repeated all analyses in a sample of participants who had MRI data for at least 1 time point. All primary caregivers provided written informed consent, and child participants provided verbal assent (younger than 12 years) or consent (12 years or older). All study procedures were reviewed and approved by the local medical ethics committee of the Erasmus MC University Medical Center.

### Physical Activity Assessments

When children were 10 years old, we used multi-informant reports (ie, primary caregiver and the child) of different dimensions of physical activity (ie, sport participation, outdoor play, and total physical activity) to comprehensively understand the associations investigated.^23^ We used both child and primary caregiver reports of physical activity separately. We also created an average measure of both reports to provide a more reliable assessment.^24^ When data were available for only 1 of the reporters, those data were also used as the multi-informant (averaged) report of physical activity. The multi-informant analyses constituted the primary analyses for hypothesis testing. The questionnaires for the primary caregiver were mostly completed by the mother (97%). Further details about the physical activity assessment are provided in eMethods 1 and eTable 1 in Supplement 1.

### Brain Morphology: T_1_-Weighted Images

**Figure 2.**
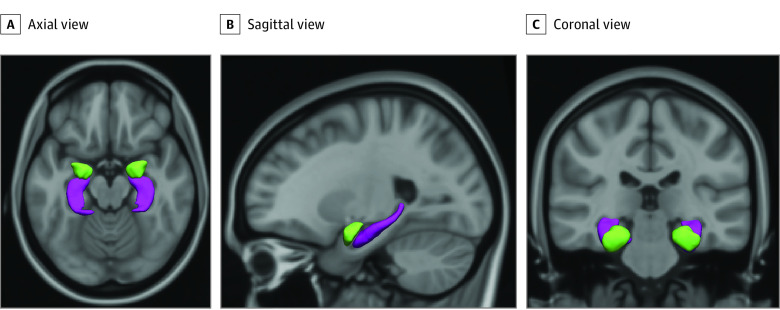
T1-Weighted Structural Magnetic Resonance Imaging (MRI) Scan (Axial, Sagittal, and Coronal View) Showing the Amygdala and Hippocampus Segmentation The amygdala is shown in green, and the hippocampus is shown in purple. Figure adapted from Cortes Hidalgo et al.^52^

High-resolution structural MRI scans were acquired using a 3-Tesla system; GE option BRAVO; repetition time, 8.77 ms; echo time, 3.4 ms; inversion time, 600 ms; flip angle, 10°; matrix size, 220 × 220; field of view, 220 mm x 220 mm; slice thickness, 1 mm; number of slices, 230; autocalibrating reconstruction for Cartesian imaging acceleration factor, 2. Data quality assurance consisted of a multistep process including both visual inspection by trained researchers and automated software.^25,26,27^ Data were processed through FreeSurfer, version 6.0 (Laboratories for Computational Neuroimaging).^28^ Briefly, nonbrain tissue was removed, voxel intensities were normalized for B1 inhomogeneity, whole brain tissue segmentation was performed, and a surface-based model of the cortex was reconstructed. Global metrics of volume were extracted (eg, subcortical gray matter volume) together with metrics of local structures (ie, hippocampus and amygdala), which were automatically labeled. We computed the total volume of hippocampus and amygdala in both hemispheres. Figure 2 illustrates the segmentation of the amygdala and hippocampus. Total intracranial volume was derived during the Talairach transformation. Further details are provided in eMethods 2 in Supplement 1.

### Assessment of Potential Confounders

According to the literature,^9^ we considered the following covariates: sex, age at MRI, maternal education, parental national origin, season of the physical activity assessment, and body mass index (BMI). Age at MRI scanning was computed according to birthdate and date of scan. Maternal education and national origin were assessed with questionnaires. Maternal educational level was categorized into high (higher vocational education and university), intermediate (secondary school, lower, or intermediate vocational training), and low (no education finished or primary school). Parental national origin was assessed through questionnaires and summarized into 3 categories: Dutch, other Western (including European, Western American, and Western Asian), and non-Western (including African, Cape Verdean, Dutch Antillean, Indonesian, Moroccan, non-Western American, non-Western Asian, Oceanian, Surinamese, and Turkish). We adjusted for parental national origin because of its potential correlation with both exposures and outcomes of the present study. Calendar season (spring, summer, autumn, and winter) was determined by the date of physical activity assessment. Height and weight were measured at age 10 years at the research center, from which BMI was calculated. For the regions of interest (namely, bilateral hippocampus and amygdala), intracranial volume (ICV) was additionally adjusted to determine whether the associations were independent of the global brain size.

### Statistical Analysis

A detailed description of our analyses is provided in eMethods 3 in Supplement 1. Analyses were run using R statistical software, version 3.4.3 (R Project for Statistical Computing). Using the lme4 package,^30^ we conducted a set of linear mixed-effects models with levels of physical activity at baseline as the independent variable and repeatedly assessed brain structure (baseline and follow-up) as the dependent variable. Separate models were run for each measure of physical activity (ie, total physical activity, sport participation, and outdoor play) and each informant with all brain structures (ie, global brain metrics, hippocampus, and amygdala). The multi-informant report of physical activity constituted the primary analysis.^24^

We tested 2 models that progressively expanded to adjust for additional confounding factors.^9^ Model 1 included the fixed effect of age at the baseline brain scan, sex, national origin, maternal education, and season of physical activity assessment. Model 2 was additionally adjusted for the fixed effects of BMI at baseline. All models included random intercepts for each participant. The models for the hippocampal and amygdala volume also included ICV (fixed effect). In contrast, we did not adjust the analyses of global brain metrics for ICV because of the high multicollinearity.^31^ All tests were 2-sided, and significance was set at a *P* value of less than .05.

Given that the prior literature does not allow us to establish specific hypotheses for left and right hemispheres, we analyzed the total volume of hippocampus and amygdala in both hemispheres. Additional hemispheric analyses are provided in eTable 2 in Supplement 1. Data were analyzed from April to December 2022.

A description of missing data are provided in eMethods 3 in Supplement 1. Missing covariate and partially incomplete physical activity data were imputed with multiple imputation by chained equations with the R package MICE.^32^ We generated 30 iterations and 30 imputed data sets; results were pooled using Rubin’s rules.^33^ In sensitivity analyses including the sample of participants who had reports of physical activity and MRI data at least 1 time point, we imputed missing data in covariates, physical activity, and MRI data.

## Results

A total of 1088 participants with repeated MRI data composed the main sample of this study; 566 participants (52%) were girls, 693 (64%) were Dutch, and the mean (SD) age of the entire cohort was 10.1 (0.6) years at baseline and 13.8 (0.5) years at follow-up. Table 1 and eTable 3 in Supplement 1 show the participants’ characteristics. A nonresponse analysis indicated no differences between participants with and without repeated MRI data. Table 2 and Table 3 demonstrate that the differences between the basic and fully adjusted models were minimal, and hence we focus on the latter. Table 2 shows no consistent association between physical activity and the change in any global brain volume measure. Significance was reached only in 2 associations for different physical activity measures, as reported only by the primary caregiver: (1) 1 hour per week more in sport participation was associated with a 64.0-mm^3^ larger volume change in subcortical gray matter (95% CI, 3.0-125.0 mm^3^; *P* = .04) and (2) 1 hour per week more in total physical activity was associated with a 154.0-mm^3^ larger volume change in total white matter (95% CI, 27.4-280.6 mm^3^; *P* = .02) from late childhood to early adolescence. Results were similar in sensitivity analyses; see eTable 4 in Supplement 1.

**Table 1.** Characteristics of Participants in the Main Sample of the Study^a^

Characteristic	Participants, No. (%)
Age at baseline, mean (SD), y	10.1 (0.6)
Age at follow-up, mean (SD), y	13.8 (0.5)
Body mass index at baseline, mean (SD)^b^	17.3 (2.4)
Sport participation at baseline, mean (SD), h/wk	
Self-reported by children	2.9 (1.4)
Reported by primary caregivers	2.8 (1.2)
Average of both reports	2.8 (1.2)
Outdoor play at baseline, mean (SD), h/wk	
Self-reported by children	7.8 (6.2)
Reported by primary caregivers	6.5 (5.1)
Average of both reports	7.1 (5.1)
Total physical activity at baseline, mean (SD), h/wk	
Self-reported by children	10.7 (6.2)
Reported by primary caregivers	9.3 (5.3)
Average of both reports	9.9 (5.3)
Sex	
Female	566 (52.0)
Male	522 (48.0)
National origin	
Dutch	693 (63.7)
Other Western^c^	108 (9.9)
Non-Western^d^	274 (25.2)
Missing data	13 (1.2)
Maternal education level	
No or only primary studies	27 (2.5)
Secondary studies or lower/intermediate vocational training	371 (34.1)
Higher vocational training or university	673 (61.8)
Missing data	17 (1.6)
Season of physical activity assessment	
Self-reported by children	
Spring	300 (27.7)
Summer	158 (14.5)
Autumn	194 (17.8)
Winter	347 (32.0)
Missing data	89 (8.0)
Reported by primary caregivers	
Spring	288 (26.5)
Summer	154 (14.2)
Autumn	257 (23.6)
Winter	369 (33.9)
Missing data	20 (1.8)

^a^ Nonimputed data are shown.

^b^ Body mass index is calculated as weight in kilograms divided by height in meters squared.

^c^ Other Western includes European, Western American, and Western Asian.

^d^ Non-Western includes African, Cape Verdean, Dutch Antillean, Indonesian, Moroccan, non-Western American, non-Western Asian, Oceanian, Surinamese, and Turkish.

**Table 2.** Associations Between Physical Activity and Longitudinal Changes in Volumes of Global Brain Metrics

Activity type, reporter, and model^a^	Changes in volume by brain region					
	Cortical gray matter, mm^3^		Subcortical gray matter, mm^3^		Total white matter, mm^3^	
	β (95% CI)^b^	*P* value^c^	β (95% CI)^b^	P value^c^	β (95% CI)^b^	*P* value^c^
Sport participation, h/wk						
Self-reported by children						
Model 1	274.8 (−537.6 to 1087.2)	.51	1.8 (−51.7 to 55.3)	.95	353.3 (−130.4 to 837)	.15
Model 2	275.2 (−537.0 to 1087.4)	.51	1.7 (−51.8 to 55.2)	.95	354.2 (−129.3 to 837.7)	.15
Reported by primary caregivers						
Model 1	313.9 (−612.6 to 1240.4)	.51	62.4 (1.4 to 123.4)	.04	197.6 (−358.7 to 753.9)	.49
Model 2	281.9 (−646.2 to 1210)	.55	64 (3 to 125)	.04	188.9 (−368.5 to 746.3)	.50
Average of both reports						
Model 1	478.6 (−415.2 to 1372.4)	.30	54.3 (−4.5 to 113.1)	.07	363.8 (−178.1 to 905.7)	.19
Model 2	449.9 (−445.2 to 1345)	.32	55.8 (−3.2 to 114.8)	.06	356.9 (−186.0 to 899.8)	.20
Outdoor play, h/wk						
Self-reported by children						
Model 1	170.2 (−7.4 to 347.8)	.06	5.8 (−6.2 to 17.8)	.34	40.9 (−67.3 to 149.1)	.46
Model 2	167.6 (−10.2 to 345.4)	.06	6.2 (−5.8 to 18.2)	.31	37.3 (−71.1 to 145.7)	.50
Reported by primary caregivers						
Model 1	137.9 (−71.6 to 347.4)	.20	7.6 (−6.3 to 21.5)	.28	112.5 (−15.9 to 240.9)	.09
Model 2	144.3 (−65.4 to 354)	.18	7.4 (−6.5 to 21.3)	.30	114 (−14.6 to 242.6)	.08
Average of both reports						
Model 1	158.1 (−50.6 to 366.8)	.14	9 (−4.9 to 22.9)	.20	107.5 (−21.3 to 236.3)	.10
Model 2	159.2 (−49.7 to 368.1)	.14	9.1 (4.8 to 23)	.20	105.5 (−23.5 to 234.5)	.11
Total physical activity, h/wk						
Self-reported by children						
Model 1	73.4 (−116.5 to 263.2)	.45	1.6 (−10.9 to 14.1)	.81	30.9 (−82.4 to 144.2)	.59
Model 2	73.2 (−116.7 to 263.1)	.45	1.8 (−10.7 to 14.3)	.78	28 (−85.3 to 141.3)	.63
Reported by primary caregivers						
Model 1	106.9 (−102.2 to 316)	.32	10.9 (−3.0 to 24.8)	.13	153 (26.6 to 279.4)	.02
Model 2	111.5 (−97.6 to 320.6)	.30	10.7 (−3.2 to 24.6)	.13	154 (27.4 to 280.6)	.02
Average of both reports						
Model 1	76.3 (−132.6 to 285.2)	.47	9.2 (−4.5 to 22.9)	.19	122.8 (−4.4 to 250)	.06
Model 2	80.4 (−128.5 to 289.3)	.45	9.1 (−4.6 to 22.8)	.19	122.5 (−4.9 to 249.9)	.06

^a^ All models were adjusted for random effects of participant. Model 1 was adjusted for the following fixed effects: age at brain scan at baseline (ie, at 10 years), sex, national origin, maternal education, and season of physical activity assessment. Model 2 was additionally adjusted for body mass index at baseline (fixed effect).

^b^ β indicates unstandardized regression coefficients.

^c^ Significance was set at *P* < .05.

**Table 3.** Associations Between Physical Activity and Longitudinal Changes in Volumes of Hippocampus and Amygdala

Activity type, reporter, and model^a^	Changes in volume by brain region			
	Hippocampus, mm^3^		Amygdala, mm^3^	
	β (95% CI)^b^	*P* value^c^	β (95% CI)^b^	*P* value^c^
Sport participation, h/wk				
Self-reported by children				
Model 1	6.6 (−4.9 to 18.1)	.26	2.3 (−6.5 to 11.1)	.62
Model 2	6.6 (−4.9 to 18.1)	.26	2.3 (−6.5 to 11.1)	.62
Reported by primary caregivers				
Model 1	10.7 (−2.2 to 23.6)	.11	10.4 (0.4 to 20.4)	.04
Model 2	11 (−2.1 to 24.1)	.11	10.4 (0.4 to 20.4)	.04
Average of both reports				
Model 1	12.2 (−0.3 to 24.7)	.05	8.5 (−1.1 to 18.1)	.09
Model 2	12.4 (−0.1 to 24.9)	.05	8.5 (−1.3 to 18.3)	.09
Outdoor play, h/wk				
Self-reported by children				
Model 1	3.2 (0.7 to 5.8)	.01	2.4 (0.4 to 4.4)	.01
Model 2	3.3 (0.8 to 5.9)	.01	2.4 (0.4 to 4.4)	.01
Reported by primary caregivers				
Model 1	−0.4 (−3.3 to 2.5)	.78	1.4 (−0.9 to 3.8)	.23
Model 2	−0.5 (−3.4 to 2.4)	.76	1.4 (−0.9 to 3.8)	.22
Average of both reports				
Model 1	2.5 (−0.4 to 5.4)	.10	2.1 (−0.1 to 4.3)	.07
Model 2	2.5 (−0.4 to 5.4)	.09	2.1 (−0.3 to 4.5)	.07
Total physical activity, h/wk				
Self-reported by children				
Model 1	3.1 (0.4 to 5.8)	.02	2.4 (0.4 to 4.4)	.02
Model 2	3.1 (0.4 to 5.8)	.02	2.4 (0.4 to 4.4)	.02
Reported by primary caregivers				
Model 1	−0.7 (−3.6 to 2.2)	.64	2.2 (−0.2 to 4.6)	.06
Model 2	−0.7 (−3.6 to 2.2)	.62	2.2 (−0.2 to 4.6)	.06
Average of both reports				
Model 1	2.1 (−0.8 to 5)	.16	2.6 (0.3 to 4.9)	.03
Model 2	2.1 (−0.8 to 5)	.16	2.6 (0.3 to 4.9)	.03

^a^ All models were adjusted for random effects of participant. Model 1 was adjusted for the following fixed effects: age at brain scan at baseline (ie, at 10 years), sex, national origin, maternal education, season of physical activity assessment, and intracranial volume. Model 2 was additionally adjusted for body mass index at baseline (fixed effect).

^b^ β indicates unstandardized regression coefficients.

^c^ Significance was set at *P* < .05.

Table 3 shows that, averaged across informants, total physical activity was not associated with an increase in hippocampal volume (β = 2.1; 95% CI, −0.8 to 5.0; *P* = .16). Total physical activity was associated with hippocampal volume increases if reported by the child (β = 3.1; 95% CI, 0.4 to 5.8; *P* = .02) but not if by their primary caregiver. Note that here the effect estimates from the analysis of outdoor play according to 1 reporter were not in the CI around the effect estimate derived from the analysis using the other reporter. Sport participation was not associated with changes in the volume of the hippocampus over time. Results were similar in sensitivity analyses; eTable 4 in Supplement 1.

Table 3 further shows that both multi-informant and child reports of total physical activity (β = 2.6; 95% CI, 0.3-4.9; *P* = .03; and β = 2.4; 95% CI, 0.4-4.4; *P* = .02) as well as child reports of outdoor play (β = 2.4; 95% CI, 0.4-4.4; *P* = .01) were associated with an increase in amygdala volume over time. In general, sport participation was not prospectively associated with changes in the volume of the amygdala. As an exception, more sport participation reported by the primary caregiver was associated with larger changes in the volume of the amygdala over time (β = 10.4; 95% CI, 0.4-20.4; *P* = .04). Results were similar in sensitivity analyses (see eTable 4 in Supplement 1). As an exception, the association of total physical activity reported by the primary caregiver with the structural development of the amygdala reached significance in sensitivity analyses.

## Discussion

This population neuroimaging study found that more physical activity in late childhood was prospectively associated with an increase in amygdala volume during a 4-year transition from late childhood (age 10 years) to early adolescence (age 14 years) in the general population. Importantly, the finding for total physical activity was robust across informants. Particularly, sport participation reported by the primary caregiver and outdoor play reported by the child were positively associated with changes in amygdala volume. The pattern of associations between physical activity and changes in the hippocampal volume was less clear in that an association was found for child-reported exposures, not for primary caregiver reports. Specifically, outdoor play and total physical activity reported by the child were associated with increases in hippocampal volume over the 4-year period. Importantly, these associations appear selective to specific subcortical regions, because we did not identify robust associations between physical activity and global measures of brain morphology, including the total volume of subcortical gray matter.

The association between physical activity and amygdala volume has scarcely been studied in humans. A previous experimental study using a rodent model showed running-induced neuroplasticity in the amygdala.^16^ Prior cross-sectional studies in the general population of children have not investigated the amygdala but focused on the hippocampus.^8,34^ Our study provides only partial support for the physical activity and hippocampal structure literature in that child-reported physical activity (ie, outdoor play and total physical activity) was associated with hippocampal volume. Although child-reported physical activity may extend longitudinally (ie, across a 4-year period from late childhood to early adolescence) to hippocampal volume in children from the general population,^8^ a similar association was not observed for primary caregiver physical activity reports. The association between outdoor play and the hippocampal volume differed if parent or child report was used. We speculate that the results using the child estimates may be more valid; parents may not be able to estimate the duration of their child’s outdoor play accurately because children become more independent during adolescence.^23^ In general, our study provides novel findings showing a longitudinal association between physical activity and subcortical volume in the amygdala and, to a lesser extent, the hippocampus. Our pattern of results implies a better understanding of physical activity is necessary to fully understand the association between this behavior with subcortical morphological changes across development.

We found that the longitudinal associations of physical activity in late childhood with changes in the volume of the amygdala and hippocampus appear to be specific. That is, physical activity was not associated with changes in global brain measures regardless of the informants and physical activity measure. Although several brain structures are still developing in children and adolescents, an increase in neuronal cell numbers (postnatal neurogenesis) has been particularly demonstrated only in the amygdala and hippocampus.^35,36^ Thus, these 2 subcortical regions may exhibit more plasticity to environmental exposures, including physical activity, during the transition from late childhood to early adolescence. Indeed, physical activity increases levels of brain-derived neurotrophic factor, which may have resulted in the potentiation and proliferation of neurons in the amygdala and hippocampus.^2,37,38^

In line with the animal literature, another potential mechanism underlying our findings is that physical activity may offer an enriched experience stimulating neural development^39,40^ of the amygdala and hippocampus among other regions, given their key roles in decision-making, spatial navigation, and inhibitory control.^1,2,41,42^ Interestingly, sport participation and outdoor play may also engage children in learning experiences involving the interrelated functions of these 2 structures.^1,2,41^ For instance, when playing team-based sports, children navigate a dynamic environment, cooperate with teammates, and make decisions including when and how to pass the ball to create optimal scoring opportunities. This enriched experience involves emotions related to success or failure depending on the effectiveness of their intended action.^43,44^

Physical activity is one of the most promising environmental exposures favorably influencing health across the lifespan.^1,2,45^ This study adds to prior literature by highlighting the neurodevelopmental benefits physical activity may have on the architecture of the amygdala and hippocampus. Public health implications would arise from future experimental research if it confirmed that higher levels of physical activity in late childhood enlarge the volumes of subcortical areas. This may mean physical activity also improves cognitive functions that are subserved by the amygdala and hippocampus such as memory,^46^ navigation,^47^ and executive functioning,^48^ benefitting academic performance.^49^ The combination of physical education, active recess, and integrating movement throughout the school day (eg, physical activity breaks in the classroom) might constitute an easy to implement public health intervention.^29^

### Strengths and Limitations

The present research has strengths. First, we studied a large, population-based sample of 1088 children, which yields more precise and generalizable findings than previous studies conducted in smaller sample sizes.^14,50^ Second, this study was longitudinal and included repeated measures of brain morphology over a 4-year period, limiting reverse causality. Third, tissue misclassification errors of the amygdala and hippocampus are unlikely to be a primary explanation for the results of the present study. Two investigators independently rated the automatic segmentations of the hippocampus and amygdala at baseline confirming only approximately 0.6% of the segmentations were problematic.^25,26,27^

Despite these strengths, several limitations should be noted. First, causal inferences from our observational findings are not possible. Future RCTs are needed to experimentally investigate the causality of the associations under study. Second, the questionnaires, although used to assess physical activity in previous studies,^9^ have not been validated. This is a common caveat in the field.^51^ Although it may be advisable to include objective measurements of physical activity in future research (eg, accelerometry), current methods of objectively measuring physical activity are accurate for estimating total physical activity but not for distinguishing between different types of activity (eg, sport participation and outdoor play). Third, we only considered the outcomes of potential confounders measured at baseline, which may not fully account for the potential influence of time-varying confounders such as BMI on the longitudinal association of physical activity with changes in brain structure. Fourth, although longitudinal, this is a unidirectional study. We did not have the data to show how the observed changes in brain development may lead to changes in physical activity.

## Conclusions

The current findings suggest more physical activity in late childhood is prospectively associated with more volume in specific subcortical structures during a 4-year transition from late childhood (10 years) to early adolescence (14 years) in the general population. Findings were robust across informants for the amygdala but less consistent for the hippocampus. These subcortical areas are characterized by plasticity in the transition from late childhood to early adolescence and underlie cognition and emotion. Importantly, the observed physical activity associations were specific to these 2 subcortical structures, as we did not identify a robust association between physical activity and global measures of brain morphology. Further experimental research corroborating the causality of our findings may inform future physical activity interventions facilitating optimal child neurodevelopment, which may lead to feasible public health as well as school-based interventions to promote brain health.

## Notes

Supplement 1. **eMethods 1. **Physical Activity Assessments **eTable 1. **Information on Specific Questions and Answer Options on the Questionnaires to Assess Physical Activity **eMethods 2. **Image Acquisition, Processing, and Quality Assurance **eMethods 3. **Analyses
eReferences
**eTable 2. **Associations Between Physical Activity and Longitudinal Changes in Volumes of Hippocampus and Amygdala per Hemisphere **eTable 3. **Characteristics of the Participants in the Sample for Sensitivity Analyses **eTable 4. **Sensitivity Analyses of the Associations Between Physical Activity and Longitudinal Changes in Volumes of Global Brain Metrics, Hippocampus, and Amygdala

Supplement 2.
Data Sharing Statement
