# Supplementary material for: Levels of Physical Activity at Age 10 Years and Brain Morphology Changes From Ages 10 to 14 Years
Source: JAMA Netw Open. 2023 Oct 5;6(10):e2333157. doi: 10.1001/jamanetworkopen.2023.33157 (PMC10556964; doi:10.1001/jamanetworkopen.2023.33157)
Supplement: Supplement 1. — eMethods 1. Physical Activity Assessments eTable 1. Information on Specific Questions and Answer Options on the Questionnaires to Assess Physical Activity eMethods 2. Image Acquisition, Processing, and Quality Assurance eMethods 3. Analyses eReferences eTable 2. Associations Between Physical Activity and Longitudinal Changes in Volumes of Hippocampus and Amygdala per Hemisphere eTable 3. Characteristics of the Participants in the Sample for Sensitivity Analyses eTable 4. Sensitivity Analyses of the Associations Between Physical Activity and Longitudinal Changes in Volumes of Global Brain Metrics, Hippocampus, and Amygdala [file jamanetwopen-e2333157-s001.pdf]

Supplemental Online Content

Estévez-López F, Dall'Aglio L, Rodriguez-Ayllon M, et al. Levels of physical activity at age 10 years and brain morphology changes from ages 10 to 14 years . *JAMA Netw Open*. 2023;6(10):e2333157.
doi:10.1001/jamanetworkopen.2023.33157

eMethods 1. Physical Activity Assessments
eTable 1. Information on Specific Questions and Answer Options on the Questionnaires to Assess Physical Activity
eMethods 2. Image Acquisition, Processing, and Quality Assurance
eMethods 3. Analyses
eReferences
eTable 2. Associations Between Physical Activity and Longitudinal Changes in Volumes of Hippocampus and Amygdala per Hemisphere
eTable 3. Characteristics of the Participants in the Sample for Sensitivity Analyses
eTable 4. Sensitivity Analyses of the Associations Between Physical Activity and Longitudinal Changes in Volumes of Global Brain Metrics, Hippocampus, and Amygdala

This supplemental material has been provided by the authors to give readers additional information about their work.

© 2023 Estévez-López F et al. *JAMA Network Open.*

eMethods 1. Physical Activity Assessments

Informants indicated both the frequency (days/week) and duration (minutes/day) of child's engagement in sport participation and outdoor play. The minutes per week spent on each activity were calculated as follows: weekly time spent on the activity = (days per week) * (minutes per day) [1]. A total physical activity score was calculated by adding the minutes of outdoor play and sport participation. In the case of sport participation, children only reported the frequency, but not the duration, of sports. Thus, to compute self-reported duration of sports, we multiply the frequency * 1 h/w because children's sports lessons usually last for 1 hour [2].

Table S1 shows the items and answer options of the questionnaires assessing physical activity. The R code for computing the total physical activity and its dimensions used in the present study (i.e., sport participation and outdoor play) is in the pages following table S1.

© 2023 Estévez-López F et al. *JAMA Network Open.*

eTable 1. Information on Specific Questions and Answer Options on the Questionnaires to Assess Physical Activity

**Questionnaire: children's self-reported physical activity**		
**Item reference in database**	**Question**	**Answers**
C1000184_cleaned	How many days a week do you play outside (think soccer or skating)?	1 = Never<br>2 = Not every week<br>3 = 1 day/week<br>4 = 2 days/week<br>5 = 3 days/week<br>6 = 4 days/week<br>7 = 5 days/week<br>8 = > 5days/week
C1000284_cleaned	When you play out, how long do you play around outside?	1 = Less than 30 min/day<br>2 = 30 to 60 min/day<br>3 = 1 to 2 hours/day<br>4 = 2 to 3 hours/day<br>5 = 3 to 4 hours/day<br>6 = > 4 hours/day
C1100184_cleaned	Do you play sports at a sports club or sports team?	0 = No<br>1 = Yes
C1100284_cleaned	How often do you play sports?	1 = Once a week<br>2 = Twice a week<br>3 = 3 times a week<br>4 = 4 times a week<br>5 = 5 times or more a week
**Questionnaire: proxy paternal-report physical activity**		
**Item reference in database**	**Question**	**Answers**
H0700281_cleaned	How many hours per week does your child spend doing sports (training and compete together)?	1 = Less than 1 hour/week<br>2 = 1 to 2 hours/week<br>3 = 2 to 4 hours/week<br>4 = More than 4 hours/week
H0800181_cleaned	On average how many days per week does your child play outside?	1 = Never<br>2 = 1 or 2 days/week<br>3 = 3 or 4 days/week<br>4 = 5 or more days/week
H0800281_cleaned	Approximately how long does your child approximately play outside per day? Only consider the days that your child plays outside	1 = Less than 30 minutes/day<br>2 = 30 minutes to 1 hour/day<br>3 = 1 to 2 hours/day<br>4 = 2 to 3 hours/day<br>5 = 3 to 4 hours/day<br>6 = More than 4 hours/day

Note. The scripts used for computing levels of physical activity are publicly available at https://github.com/FerEstevezLopez/doi_10.1001-jamanetworkopen.2023.33157

© 2023 Estévez-López F et al. *JAMA Network Open.*

eMethods 2. Image Acquisition, Processing, and Quality Assurance

All children first underwent a mock scanning session to become acquainted with the MRI scanning environment. Structural T1 MRI scans were obtained on a 3-Tesla scanner (Discovery MR-750W, General Electric Worldwide, Milwaukee, WI, USA). We used an 8-channel head coil to obtain inversion recovery fast spoiled gradient recalled sequence (IR-FSPGR) images of the whole-brain at high resolution. GE option BRAVO, TR = 8.77ms, TE = 3.4ms, TI = 600ms, Flip Angle = 10º, Matrix Size = 220 x 220, Field of View = 220mm x 220mm, slice thickness = 1mm, number of slices = 230, ARC acceleration factor = 2.

Preprocessing of the T1-weighted images is performed using the FreeSurfer Software, version 6.0 (http://surfer.nmr.mgh.harvard.edu). In brief, processing includes intensity normalization, skull stripping, Talairach transformation, segmentation of the white and gray matter structures, tessellation of the gray-white matter boundary, topology correction, and surface deformation to identify the gray-white matter boundary and the gray-cerebrospinal fluid boundary.

Following preprocessing with FreeSurfer, images were visually inspected to assess whether white and pial surfaces followed proper boundaries. Cortical thickness was calculated as the closest distance from the gray-white matter boundary to the gray-cerebrospinal fluid boundary at each vertex on the tessellated surface. Total cortical surface area was calculated per hemisphere by summing the areas of each triangular isosurface that layered the WM/GM interface. Subcortical volumes are calculated from FreeSurfer's automated segmentation procedure.

For the subcortical analyzes, segmentation of hippocampus and amygdala occurred according to the preprogrammed atlas in FreeSurfer. Total intracranial volume was extracted using the same segmentation pipeline.

For quality checking of the subcortical segmentations, statistical outliers were determined by calculating the interquartile range for each of the volumes. Unnaturally low or high values would be excluded, but this was not the case in any segmentations.

© 2023 Estévez-López F et al. *JAMA Network Open.*

eMethods 3. Analyses

The main analyses comprised linear mixed-effects models, which are widely used in longitudinal research because of their flexibility in modelling repeated measures data and the ability to incorporate both fixed and random effects[3,4] Thus, our models included a random intercept to explicitly account for within-individual associations between repeated measurements of brain structure as a random effect, which has been suggested to be more advantageous than traditional approaches (e.g., linear regression adjusting for baseline levels of the outcome).[5] Analyses were run using the R statistical software (version 3.4.3), using the *lme4* package[6] for running linear mixed-effects models.

Our linear mixed-effects models included levels of physical activity at baseline as the independent variable and repeatedly assessed brain structure (baseline and follow-up) as the dependent variable. Separate models were run for each measure of physical activity (i.e., total physical activity, sport participation, and outdoor play) and each informant with all brain structures (i.e., global brain metrics, hippocampus and amygdala). Here, we chose the multi-informant report of physical activity for the primary analyses given the more robust assessment[7].

We tested two models that progressively expanded to adjust for additional confounding factors selected based on prior literature.[8] Model 1 included the fixed effect of age at the baseline brain scan, sex, national origin, and maternal education. Model 2 was additionally adjusted for the fixed effects of BMI at baseline. BMI was not included in Model 1 because its role (e.g., confounder, mediator or collider) in the association between physical activity and brain morphology is unclear. Importantly, adjusting for non-cofounding variables may introduce bias in the results.[9] All models included random intercepts for each participant. The models for the hippocampal and amygdala volume

© 2023 Estévez-López F et al. *JAMA Network Open.*

also included ICV (fixed effect). In contrast, we did not adjust the analyses of global brain metrics for ICV because of the high multicollinearity[10].

The formula of our model was: $Brain_{ij} \sim \beta_0 + \beta physical\ activity_i * time_{ij} + \beta physical\ activity_i + \beta age_i + \beta sex_i + \beta national\ origin_i + \beta maternal\ education_i + \beta season\ of\ physical\ activity\ assessment_i, \beta BMI_i$ + (1|Particpant ID); where *i* is the individual, *j* is the time point, *Brain* is volume, β_0 is the intercept, β is the regression coefficient, *physical activity* is a continuous score, *time* is a factor either baseline or follow-up, *sex, national origin, maternal education, season of physical activity assessment* are factors, *BMI* is a continuous score -included in Model 2 only- and *(1|Particpant ID)* is the random effect for individuals. Analyses for hippocampus and amygdala, also included *ICV* as a continuous score. All the coefficients reported in the manuscript are the $\beta physical\ activity_i * time_{ij}$ term, thus, for every unit change in *physical activity* per average follow-up time (4 years) is related to $\beta physical\ activity_i * time_{ij}$ change in brain volume.

Given that the prior literature does not allow us to establish specific hypotheses for left and right hemispheres, we analysed the total volume of hippocampus and amygdala in both hemispheres. Additional hemispheric analyses are provided in eTable 2 in the Supplement.

Missing data

Missing data were observed for national origin, maternal education, BMI (all, ≤1%) and season of the physical activity assessment (8% for self-reports and 2% for primary caregivers). In the primary analyses (1,088 participants with repeated MRI data), missing data for one of the physical activity assessments were observed as follows: child reports of sport participation (12%), child reports of outdoor play (3%), primary caregiver reports

© 2023 Estévez-López F et al. *JAMA Network Open.*

of sport participation (8%), and primary caregiver reports of outdoor play (4%). Missing covariate and physical activity data were imputed with Multiple Imputation by Chained Equations with the R package MICE[11]. We generated 30 iterations and 30 imputed datasets, across which results were pooled using Rubin's rules[12].

In sensitivity analyses including the sample of 3,459 participants who had reports of physical activity and MRI data at least one-time point, we imputed missing data in covariates, physical activity, and MRI data.

eReferences

1. Rodriguez-Ayllon M, Derks IPM, van den Dries MA, et al. Associations of physical activity and screen time with white matter microstructure in children from the general population. *Neuroimage*. 2020;205:116258. doi:10.1016/j.neuroimage.2019.116258
2. Vasconcellos F, Seabra A, Cunha F, et al. Health markers in obese adolescents improved by a 12-week recreational soccer program: a randomised controlled trial. *J Sports Sci*. 2016;34(6):564-575. doi:10.1080/02640414.2015.1064150
3. Roberts AL, Liu J, Lawn RB, et al. Association of Posttraumatic Stress Disorder with Accelerated Cognitive Decline in Middle-aged Women. *JAMA Netw Open*. 2022;5(6):E2217698. doi:10.1001/jamanetworkopen.2022.17698
4. Zhang W, Singh SP, Clement A, Calfee RP, Bijsterbosch JD, Cheng AL. Improvements in Physical Function and Pain Interference and Changes in Mental Health Among Patients Seeking Musculoskeletal Care. *JAMA Netw Open*. 2023;6(6):e2320520. doi:10.1001/jamanetworkopen.2023.20520
5. Seltman HJ. Mixed Models: A flexible approach to correlated data. In: *Experimental Design and Analysis*. ; 2014:357-378.
6. Bates D, Mächler M, Bolker B, Walker S. Fitting Linear Mixed-Effects Models Using lme4. *J Stat Softw*. 2015;67(1). doi:10.18637/jss.v067.i01
7. Chaumeton N, Duncan SC, Duncan TE, Strycker LA. A measurement model of youth physical activity using pedometer and self, parent, and peer reports. *Int J*

© 2023 Estévez-López F et al. *JAMA Network Open.*

Behav Med. 2011;18(3):209-215. doi:10.1007/s12529-010-9118-5

8. Rodriguez-Ayllon M, Neumann A, Hofman A, et al. Neurobiological, Psychosocial, and Behavioral Mechanisms Mediating Associations between Physical Activity and Psychiatric Symptoms in Youth in the Netherlands. *JAMA Psychiatry*. 2023;80(5):451-458. doi:10.1001/jamapsychiatry.2023.0294
9. Dall'aglio L, Kim HH, Lamballais S, Labrecque J, Muetzel RL, Tiemeier H. Attention-deficit hyperactivity disorder symptoms and brain morphology: Examining confounding bias. *Elife*. 2022;11. doi:10.7554/eLife.78002
10. Kocevska D, Muetzel RL, Luik AI, et al. The Developmental Course of Sleep Disturbances Across Childhood Relates to Brain Morphology at Age 7: The Generation R Study. *Sleep*. 2017;40(1). doi:10.1093/sleep/zsw022
11. van Buuren S, Groothuis-Oudshoorn K. mice: Multivariate imputation by chained equations in R. *J Stat Softw*. 2011;45(3):1-67. doi:10.18637/jss.v045.i03
12. Rubin D. *Multiple Imputation for Nonresponse in Surveys*. John Wiley & Sons; 2004.

© 2023 Estévez-López F et al. *JAMA Network Open.*

eTable 2. Associations Between Physical Activity and Longitudinal Changes in Volumes of Hippocampus and Amygdala per Hemisphere

**Changes in the volume of:**	**Hippocampus (mm^3)**						**Amygdala (mm^3)**					
	**Left hemisphere**			**Right hemisphere**			**Left hemisphere**			**Right hemisphere**		
**Sport participation (hours/week)**	**B**	**SE**	**p**	**B**	**SE**	**p**	**B**	**SE**	**p**	**B**	**SE**	**p**
Self-reported by children (*n*=987)												
Model 1	3.3	3.6	0.4	3.3	3.5	0.3	-1.3	2.8	0.6	3.6	2.9	0.2
Model 2	3.3	3.6	0.4	3.3	3.5	0.3	-1.3	2.8	0.6	3.6	2.9	0.2
Reported by primary caregivers (*n*=1,052)												
Model 1	3.7	4.0	0.4	7.0	3.9	0.07	4.6	3.2	0.2	5.8	3.3	0.08
Model 2	4.1	4.0	0.3	6.9	3.9	0.08	4.5	3.2	0.2	5.9	3.3	0.08
Average of both reports (*n*=1,088)												
Model 1	3.5	3.9	0.4	6.3	3.8	0.1	2.9	3.1	0.4	4.9	3.2	0.1
Model 2	3.9	3.9	0.3	6.1	3.8	0.1	2.8	3.1	0.4	5.0	3.2	0.1
**Outdoor play (hours/week)**	**B**	**SE**	**p**	**B**	**SE**	**p**	**B**	**SE**	**p**	**B**	**SE**	**p**
Self-reported by children (*n*=987)												
Model 1	2.2	0.8	**0.004**	1.0	0.8	0.2	0.7	0.6	0.3	1.7	0.6	**0.006**
Model 2	2.3	0.8	**0.003**	1.0	0.8	0.2	0.7	0.6	0.3	1.7	0.6	**0.006**
Reported by primary caregivers (*n*=1,052)												
Model 1	-0.3	0.9	0.7	-0.1	0.9	0.9	0.3	0.7	0.7	1.1	0.8	0.1
Model 2	-0.4	0.9	0.7	-0.1	0.9	0.9	0.3	0.7	0.7	1.1	0.8	0.1
Average of both reports (*n*=1,088)												
Model 1	1.5	0.9	0.1	1.0	0.9	0.2	0.5	0.7	0.4	1.8	0.7	**0.01**
Model 2	1.4	0.9	0.2	1.1	0.9	0.2	0.5	0.7	0.4	1.8	0.7	**0.01**
**Total physical activity (hours/week)**	**B**	**SE**	**p**	**B**	**SE**	**p**	**B**	**SE**	**p**	**B**	**SE**	**p**

© 2023 Estévez-López F et al. *JAMA Network Open.*

Self-reported by children (*n*=987)												
Model 1	2.1	0.8	**0.01**	1.0	0.8	0.2	0.5	0.7	0.4	1.9	0.7	**0.005**
Model 2	2.1	0.8	**0.01**	1.0	0.8	0.2	0.5	0.7	0.4	1.9	0.7	**0.005**
Reported by primary caregivers (*n*=1,052)												
Model 1	-0.5	0.9	0.6	-0.2	0.9	0.8	0.7	0.7	0.3	1.5	0.8	**0.048**
Model 2	-0.6	0.9	0.5	-0.2	0.9	0.9	0.7	0.7	0.3	1.5	0.8	**0.049**
Average of both reports (*n*=1,088)												
Model 1	1.1	0.9	0.2	0.8	0.9	0.3	0.6	0.7	0.4	2.1	0.7	**0.005**
Model 2	1.1	0.9	0.2	0.9	0.9	0.3	0.6	0.7	0.4	2.1	0.7	**0.005**

Note. Significant p-values (i.e., < 0.05) are indicated in bold. B, unstandardized regression coefficients. SE, standard error. All models were adjusted for random effects of participant. Model 1 was adjusted for the following fixed effects: age at brain scan at baseline (i.e., at 10 years old), sex, national origin, maternal education, and intracranial volume. Model 2 was additionally adjusted for body mass index at baseline (fixed effect).

© 2023 Estévez-López F et al. *JAMA Network Open.*

eTable 3. Characteristics of the Participants in the Sample for Sensitivity Analyses (*n*=3,459)

	Sample participating at baseline (*n*=2,765)		Sample participating at follow up (*n*=1,782)	
	Mean	SD	Mean	SD
Age (years old) at baseline	10.1	0.6	10.1	0.7
Age (years old) at follow up	13.8	0.5	14.0	0.6
Body mass index (kg/m^2) at baseline	17.4	2.5	17.4	2.5
Sport participation at baseline				
Self-reported by children (hours/week)	2.9	1.5	2.9	1.4
Reported by primary caregivers (hours/week)	2.8	1.2	2.8	1.2
Average of both reports (hours/week)	2.8	1.2	2.8	1.2
Outdoor play at baseline				
Self-reported by children (hours/week)	7.8	6.2	7.7	6.2
Reported by primary caregivers (hours/week)	6.8	5.3	6.6	5.2
Average of both reports (hours/week)	7.2	5.2	7.1	5.2
Total physical activity at baseline				
Self-reported by children (hours/week)	10.7	6.4	10.8	6.3
Reported by primary caregivers (hours/week)	9.6	5.5	9.4	5.4
Average of both reports (hours/week)	10.0	5.5	9.9	5.4
	N	%	N	%
Sex				
Female	1,393	50.4	954	53.5
Male	1,372	49.6	828	46.5
National origin				
Dutch	1,773	64.1	1,138	63.8
Other western	251	9.1	175	9.8
Non-western	714	25.8	452	25.4
Missing data	27	1.0	17	1.0
Maternal education level				
No/Primary/Secondary studies	1,832	66.3	1,175	66.0
Higher education	899	32.5	585	32.8
Missing data	34	1.2	22	1.2

Note. Non-imputed data are shown. SD, standard deviation.

© 2023 Estévez-López F et al. *JAMA Network Open.*

eTable 4. Sensitivity Analyses of the Associations Between Physical Activity and Longitudinal Changes in Volumes of Global Brain Metrics, Hippocampus, and Amygdala

**Changes in the volume of:**	**Cortical gray matter (mm^3)**			**Subcortical gray matter (mm^3)**			**Total white matter (mm^3)**			**Hippocampus (mm^3)**			**Amygdala (mm^3)**		
**Sport participation (hours/week)**	**B**	**SE**	**p**	**B**	**SE**	**p**	**B**	**SE**	**p**	**B**	**SE**	**p**	**B**	**SE**	**p**
Self-reported by children (*n*=3,058)															
Model 1	295.3	405.5	0.5	5.5	27.0	0.8	360.7	244.6	0.1	7.9	5.8	0.2	3.8	4.4	0.4
Model 2	304.5	405.3	0.5	5.7	27.0	0.8	364.9	244.6	0.1	8.0	5.8	0.2	4.0	4.4	0.4
Reported by primary caregivers (*n*=3,317)															
Model 1	310.5	462.5	0.5	65.1	30.7	**0.03**	203.7	281.3	0.5	12.3	6.6	0.06	11.4	5.0	**0.02**
Model 2	299.1	463.3	0.5	67.0	30.8	**0.03**	198.2	281.9	0.5	12.7	6.6	0.05	11.7	5.0	**0.02**
Average of both reports (*n*=3,459)															
Model 1	325.4	441.9	0.5	42.2	29.6	0.2	323.7	272.0	0.2	11.0	6.3	0.08	8.9	4.8	0.06
Model 2	310.6	442.6	0.5	43.9	29.6	0.1	318.6	272.6	0.2	11.5	6.3	0.07	9.1	4.8	0.06
**Outdoor play (hours/week)**	**B**	**SE**	**p**	**B**	**SE**	**p**	**B**	**SE**	**p**	**B**	**SE**	**p**	**B**	**SE**	**p**
Self-reported by children (*n*=3,058)															
Model 1	154.0	88.7	0.08	6.9	6.0	0.3	41.8	54.7	0.4	3.4	1.3	**0.01**	2.6	1.0	**0.01**
Model 2	154.2	88.8	0.08	7.3	6.0	0.2	39.1	54.8	0.5	3.4	1.3	**0.01**	2.6	1.0	**0.01**
Reported by primary caregivers (*n*=3,317)															
Model 1	88.1	104.6	0.4	5.0	7.0	0.5	95.3	64.9	0.1	-0.3	1.5	0.9	1.4	1.1	0.2
Model 2	97.1	104.7	0.4	5.0	7.0	0.5	98.3	65.0	0.1	-0.3	1.5	0.8	1.5	1.1	0.2
Average of both reports (*n*=3,459)															
Model 1	135.7	102.6	0.2	9.2	6.9	0.2	94.4	64.0	0.1	2.6	1.5	0.07	2.5	1.1	**0.02**
Model 2	141.3	102.6	0.2	9.5	6.9	0.2	94.5	64.1	0.1	2.7	1.5	0.07	2.5	1.1	**0.02**

© 2023 Estévez-López F et al. *JAMA Network Open.*

**Total physical activity (hours/week)**	**B**	**SE**	**p**	**B**	**SE**	**p**	**B**	**SE**	**p**	**B**	**SE**	**p**	**B**	**SE**	**p**
Self-reported by children (*n*=3,058)															
Model 1	65.4	94.8	0.5	2.8	6.3	0.7	31.1	57.3	0.6	3.4	1.3	**0.01**	2.8	1.0	**0.01**
Model 2	68.4	94.8	0.5	3.1	6.3	0.6	29.2	57.3	0.6	3.4	1.3	**0.01**	2.8	1.0	**0.01**
Reported by primary caregivers (*n*=3,317)															
Model 1	76.2	104.4	0.5	9.6	7.0	0.2	145.2	63.9	**0.02**	-0.4	1.5	0.8	2.3	1.1	**0.048**
Model 2	82.3	104.4	0.4	9.5	7.0	0.2	147.3	64.0	**0.02**	-0.5	1.5	0.8	2.3	1.1	**0.045**
Average of both reports (*n*=3,459)															
Model 1	57.5	102.6	0.6	9.1	6.8	0.2	112.6	63.3	0.08	2.2	1.5	0.1	2.9	1.1	**0.01**
Model 2	64.1	102.6	0.5	9.1	6.9	0.2	113.5	63.4	0.07	2.2	1.5	0.1	3.0	1.1	**0.01**

Note. Significant p-values (i.e., < 0.05) are indicated in bold. B, unstandardized regression coefficients. SE, standard error. All models were adjusted for random effects of participant. Model 1 was adjusted for the following fixed effects: age at brain scan at baseline (i.e., at 10 years old), sex, national origin, and maternal education. For the hippocampus and amygdala, model 1 was also adjusted for intracranial volume at baseline (fixed effect). Model 2 was additionally adjusted for body mass index at baseline (fixed effect).

© 2023 Estévez-López F et al. *JAMA Network Open.*
