# Supplementary material for: Levels of Physical Activity at Age 10 Years and Brain Morphology Changes From Ages 10 to 14 Years
Source: JAMA Netw Open. 2023 Oct 5;6(10):e2333157. doi: 10.1001/jamanetworkopen.2023.33157 (PMC10556964; doi:10.1001/jamanetworkopen.2023.33157)
Supplement: Supplement 2. — Data Sharing Statement [file jamanetwopen-e2333157-s002.pdf]

Data Sharing Statement

Estévez-López. Levels of Physical Activity at Age 10 Years and Brain Morphology Changes From Ages 10 to 14 Years. *JAMA Netw Open*. Published October 05, 2023. doi:10.1001/jamanetworkopen.2023.33157

Data

Data available: Data The Generation R data sets generated or analyzed during the current study are not publicly available due to legal and ethical regulations but may be made available upon request to the Director of the Generation R Study, Vincent Jaddoe (v.jaddoe@erasmusmc.nl), in accordance with the local, national, and European Union regulations. Dr. Fernando Estévez-López had full access to all the data in the study and takes responsibility for the integrity of the data and the accuracy of the data analysis. The scripts used for running the statistical analyses are publicly available at https://github.com/FerEstevezLopez/doi_10.1001-jamanetworkopen.2023.33157
